# Tumor Hypoxia on ^18^F-fluoromisonidazole Positron Emission Tomography and Distant Metastasis From Head and Neck Squamous Cell Carcinoma

**DOI:** 10.1001/jamanetworkopen.2024.36407

**Published:** 2024-09-30

**Authors:** Chengcheng Gui, Rick Wray, Heiko Schöder, Joseph O. Deasy, Milan Grkovski, John L. Humm, Richard J. Wong, Eric J. Sherman, Nadeem Riaz, Nancy Y. Lee

**Affiliations:** 1 Department of Radiation Oncology, Memorial Sloan Kettering Cancer Center, New York, New York; 2 Department of Radiology, Memorial Sloan Kettering Cancer Center, New York, New York; 3 Department of Medical Physics, Memorial Sloan Kettering Cancer Center, New York, New York; 4 Department of Surgery, Memorial Sloan Kettering Cancer Center, New York, New York; 5 Department of Medicine, Memorial Sloan Kettering Cancer Center, New York, New York; 6 Immunogenomics and Precision Oncology Platform, Memorial Sloan Kettering Cancer Center, New York, New York

## Abstract

**Importance:**

Given high rates of locoregional control after definitive management of head and neck squamous cell carcinoma (HNSCC), better methods are needed to project distant metastasis (DM) risk. Tumor hypoxia on ^18^F-fluoromisonidazole (FMISO) positron emission tomography (PET) is associated with locoregional failure, but data demonstrating an association with DM are limited.

**Objective:**

To determine whether tumor hypoxia on FMISO PET is associated with DM risk after chemoradiotherapy (CRT) for HNSCC.

**Design, Setting, and Participants:**

This cohort study assessed patients with HNSCC enrolled in 2 prospective clinical trials at a single academic referral center from 2004 to 2021 in which participants received FMISO PET before and during CRT. Data analysis occurred from May 2023 to May 2024.

**Exposures:**

FMISO PET scans before and 1 to 2 weeks after starting CRT were evaluated for tumor hypoxia by nuclear medicine physicians.

**Main Outcomes and Measures:**

The primary outcome was DM, defined as biopsy-proven HNSCC outside the primary site and regional lymph nodes. Time to DM was modeled with competing risk regression, with death as a competing risk. Overall survival (OS) was assessed secondarily and modeled with Cox regression.

**Results:**

Among 281 patients (median [range] age at CRT, 58.7 [25.5-85.6] years; 251 male [89.3%]) included in this study, 242 (86.1%) had oropharyngeal primary cancer, and 266 (94.7%) had human papillomavirus–positive disease. Of all patients, 217 (77.2%) had T stage 1 or 2, and 231 patients (82.2%) had N stage 2b or less. De-escalated 30 Gy CRT was delivered to 144 patients (51.2%), and the remainder received standard 70 Gy CRT. On FMISO PET examination, 73 patients (26.0%) had hypoxia-negative disease before CRT, 138 patients (49.1%) had hypoxia-positive disease before CRT and then hypoxia-negative disease during CRT, and 70 patients (24.9%) persistently had hypoxia-positive disease before and during CRT. At a median (IQR) 58 (46-91) months of follow-up, 12 DM events and 22 deaths were observed. Persistent intratreatment hypoxia was associated with increased DM risk (hazard ratio, 3.51; 95% CI, 1.05-11.79; *P* = .04) and worse OS (hazard ratio, 2.66; 95% CI, 1.14-6.19; *P* = .02). No patients with hypoxia-negative disease before CRT experienced DM.

**Conclusions and Relevance:**

In this cohort study using pooled analysis of prospective nonrandomized clinical trials incorporating FMISO PET in the definitive management of HNSCC, persistent intratreatment hypoxia was associated with increased risk of DM and worse OS. Conversely, all patients with hypoxia-negative disease before treatment remained free of DM. These findings suggest that pretreatment and intratreatment FMISO PET results may serve as biomarkers for DM risk and aid in identifying candidates for escalated therapeutic strategies.

## Key Points

**Question:**

Is tumor hypoxia on pretreatment or intratreatment ^18^F-fluoromisonidazole positron emission tomography (PET) associated with distant metastasis after chemoradiotherapy for head and neck squamous cell carcinoma (HNSCC)?

**Findings:**

In this cohort study of 281 patients, persistent tumor hypoxia on intratreatment 18F-fluoromisonidazole PET was associated with a 3.5-fold increased risk of distant metastasis after chemoradiotherapy. Conversely, all patients who were negative for tumor hypoxia on pretreatment ^18^F-fluoromisonidazole PET remained free of distant metastasis .

**Meaning:**

These findings suggest that hypoxia on ^18^F-fluoromisonidazole PET may serve as a clinical biomarker of distant metastasis risk for patients with HNSCC who are treated with chemoradiotherapy and guide patient selection for escalated therapeutic strategies.

## Teaser

This cohort study assesses whether tumor hypoxia on ^18^F-fluoromisonidazole positron emission tomography (PET) scan is associated with distant metastasis among patients with head and neck squamous cell carcinoma (SCC).

## Introduction

Long-term survival of patients with head and neck squamous cell carcinoma (HNSCC) remains approximately 50% to 60% across all subsites and exceeds 75% for human papillomavirus (HPV)-positive disease.^1,2^ While chemoradiotherapy (CRT) improves locoregional control,^3^ distant metastasis (DM) occurs in 10% to 15% of patients, with poor prognosis.^1,2^ Novel biomarkers are needed to identify patients at high risk of DM who may benefit from escalated therapeutic strategies, including novel chemotherapy and immunotherapy regimens.^4^ This is particularly important in patients with early-stage disease, for whom risk of locoregional failure is low.

Tumor hypoxia is associated with resistance to CRT and propensity for metastasis in preclinical models and clinical data across many cancer types.^5,6,7^ While clinical assessment of tumor hypoxia has historically been challenging, ^18^F-fluoromisonidazole (FMISO) positron emission tomography (PET) has emerged as a method for reliable, noninvasive measurement.^8,9,10,11,12,13^

In HNSCC, tumor hypoxia on FMISO is associated with locoregional failure after CRT.^14,15,16,17,18^ While limited evidence suggests that hypoxia on FMISO PET is also associated with DM, consistent with preclinical models, this association is inadequately explored.^19,20^ To address this knowledge gap, we pooled data from prospective nonrandomized clinical trials to perform what is, to our knowledge, the largest analysis of tumor hypoxia on FMISO PET as a biomarker of DM risk after CRT for HNSCC.

## Methods

### Study Design and Participants

This cohort study was approved by the Memorial Sloan Kettering Cancer Center institutional review board and followed Strengthening the Reporting of Observational Studies in Epidemiology (STROBE) reporting guideline. Patients with nonmetastatic HNSCC preparing to undergo CRT as part of definitive management at a single academic institution were enrolled in 2 prospective phase II studies that incorporated FMISO PET (Lee et al^16^ [2016], Riaz et al,^17^ and Lee et al^18^ [2024]), reported previously. Written informed consent was obtained from all patients who enrolled. Patients treated on these studies between August 2004 and January 2021 were considered for inclusion in this unplanned secondary analysis. Lee et al (2016)^16^ and Riaz et al,^17^ which enrolled patients from 2004 to 2016, included patients with HPV-positive and HPV-negative HNSCC with various primary sites. Lee et al (2024)^18^ cohort A, which enrolled patients from 2017 to 2021, included HPV-positive HNSCC of the oropharynx or unknown primary site, T stage 0 to 2, and N stage 1 to 2c per American Joint committee on Cancer (AJCC), seventh edition staging. In this protocol, oropharyngeal primary cancers were resected before CRT, although a negative margin was not required. FMISO PET imaging before and 1 to 2 weeks after starting CRT were required for inclusion. Completion of the planned course of CRT was required for inclusion. A flow diagram illustrating the number of patients included from each of the prospective studies is provided (eFigure 1 in Supplement 1). Tumor hypoxia status was evaluated by nuclear medicine physicians. Protocols for obtaining and interpreting FMISO PET are described previously.^12,16,17,18^ Patients with oropharyngeal primary cancers who were negative for intratreatment hypoxia were eligible for 30 Gy de-escalated CRT.

The primary outcome was time to DM from CRT completion. DM was identified as biopsy-proven HNSCC outside the primary site and regional lymph nodes. The date of DM was based on the earliest imaging study in which the biopsied metastatic lesion was identifiable.

### Statistical Analysis

DM, with death as a competing risk, was assessed with cumulative incidence estimation, Gray test, and the Fine-Gray subdistribution hazard model. Patients were censored at the date of the last computed tomography study encompassing the chest, abdomen, and pelvis. Overall survival (OS) was assessed as a secondary outcome. OS was assessed with the Kaplan-Meier method and Cox proportional hazards model. Patients were censored at the last date on which they were known to be alive.

Univariable model hazard ratios (HRs) are reported for selected baseline characteristics. Multivariable models were not evaluated given few DM and OS events. Associations of hypoxia with other baseline characteristics were evaluated with univariable logistic regression and Fisher exact test. Statistical significance was defined as *P* < .05. Analyses were conducted using R statistical software version 4.3.0 (R Project for Statistical Computing). Analysis was conducted from May 2023 to May 2024.

## Results

Baseline characteristics of the 281 patients (median [range] age at CRT, 58.7 [25.5-85.6] years; 251 male [89.3%]) are summarized in Table 1. Most patients had primary oropharyngeal tumors (242 patients [86.1%]) and HPV-positive disease by p16 immunohistochemistry or RNA in situ hybridization (266 patients [94.7%]). Most patients had early-stage disease, including 217 patients (77.2%) with T stage 1 or 2 and 231 patients (82.2%) with N stage 2b or less. Approximately one-half of patients had primary tumor resection before CRT (148 patients [52.7%]). Approximately one-half of patients received 30 Gy de-escalated CRT (144 patients [51.2%]), and the remainder received standard 70 Gy CRT. Most patients received cisplatin-based chemotherapy (239 patients [85.1%]).

**Table 1.** Patient, Disease, and Treatment Characteristics

Characteristic	Patients, No. (%) (N =281)
Clinical trial	
Lee et al, 2016^16^ and Riaz et al, 2021^17^	129 (45.9)
Lee et al 2024^18^	152 (54.1)
Age at chemoradiotherapy, median (range), y	58.7 (25.5-85.6)
Sex	
Male	251 (89.3)
Female	30 (10.7)
Karnofsky performance status score <80	5 (1.8)
Smoking history	
At least 1 pack-year	124 (44.1)
Ongoing at time of chemoradiation	13 (4.6)
Primary site	
Oropharynx	242 (86.1)
Base of tongue	100 (35.6)
Tonsil	142 (50.5)
Oral cavity	1 (0.4)
Hypopharynx	3 (1.1)
Larynx	3 (1.1)
Unknown	32 (11.4)
HPV status^a^	
Positive	266 (94.7)
Negative	11 (3.9)
Unknown	4 (1.4)
T stage	
T0	32 (11.4)
T1	108 (38.4)
T2	109 (38.8)
T3	21 (7.5)
T4	11 (3.9)
N stage	
N0	4 (1.4)
N1	36 (12.8)
N2a	28 (10.0)
N2b	163 (58)
N2c	48 (17.1)
N3	2 (0.7)
Primary tumor resection before chemoradiotherapy	148 (52.7)
Radiotherapy regimen	
Standard (70 Gy in 35 fractions)	137 (48.8)
De-escalated (30 Gy in 15 fractions)	144 (51.2)
Concurrent systemic therapy regimen	
Cisplatin-based	239 (85.1)
Platinum-based	273 (97.2)
Cetuximab only	4 (1.4)
Hypoxia on FMISO PET	
Negative pretreatment	73 (26.0)
Positive pretreatment, negative intratreatment	138 (49.1)
Positive pretreatment, positive intratreatment	70 (24.9)

Abbreviation: FMISO PET, ^18^F-fluoromisonidazole positron emission tomography; HPV, human papillomavirus.

^a^ Determined by p16 immunohistochemistry or HPV in situ hybridization.

FMISO PET showed that 73 patients (26.0%) had hypoxia-negative disease before CRT, 138 patients (49.1%) had hypoxia-positive disease before CRT and subsequently hypoxia-negative disease during CRT, and 70 patients (24.9%) persistently had hypoxia-positive disease before and during CRT. Representative FMISO PET imaging of patients in each of the 3 categories is provided in eFigure 2 in Supplement 1. N stage 2c or greater was associated with persistent intratreatment hypoxia (odds ratio, 2.40; 95% CI, 1.19-4.81; *P* = .01).

Characteristics of 12 patients who experienced DM are summarized in the eTable in Supplement 1. Of the 12 patients, 4 (33.3%) experienced locoregional recurrence prior to DM. Metastatic sites included the lungs (11 patients [91.7%]), bone (3 patients [25.0%]), and liver (2 patients [16.7%]). Median (IQR) time to latest radiographic follow-up among censored patients was 24 (12-34) months. Cumulative incidence estimates of DM at 2 years were 10.2% (95% CI, 3.6%-20.7%) among patients with persistent intratreatment hypoxia and 2.4% (95% CI, 0.8%-5.7%) among those without (HR, 3.51; 95% CI, 1.05-11.79; *P* = .04) (Figure, A). Among cases with hypoxia-negative disease before CRT, none experienced DM (Gray test *P* = .03) (Figure, B). HPV-positive disease was associated with lower risk of DM (HR, 0.13; 95% CI, 0.02-0.92; *P* = .04) (Table 2).

**Figure.**
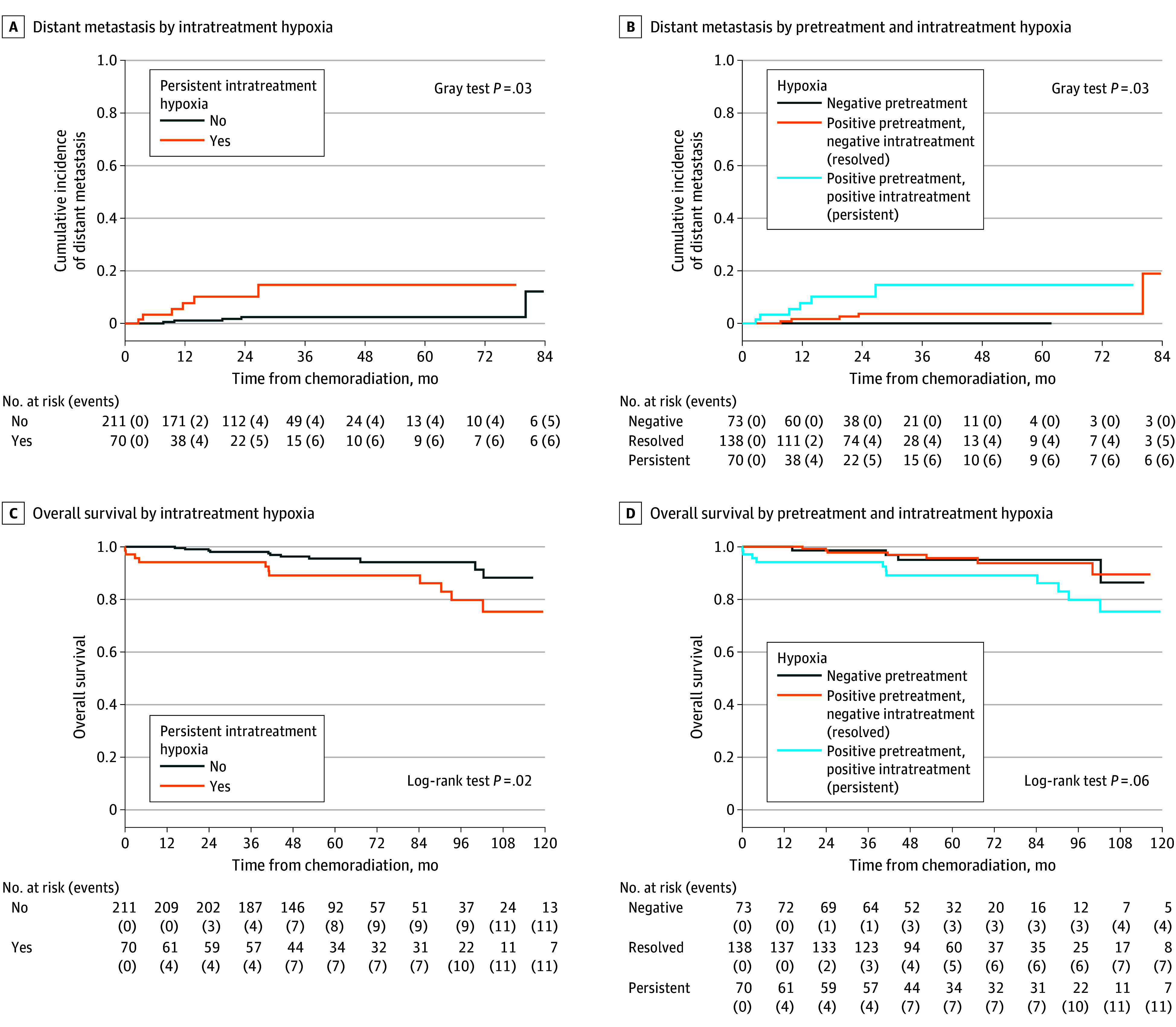
Distant Metastasis and Overall Survival After Chemoradiotherapy by Pretreatment and Intratreatment Hypoxia Status

**Table 2.** Univariable Associations Between Baseline Characteristics and Outcomes

Characteristic	Distant metastasis^a^		Overall survival^b^	
	HR (95% CI)	*P* value	HR (95% CI)	*P* value
Age at chemoradiation	1.03 (0.94-1.13)	.53	1.13 (1.08-1.19)	<.001
Smoking history 1 pack-year or greater	1.25 (0.43-3.68)	.68	1.67 (0.71-3.92)	.24
Oropharyngeal primary	0.74 (0.16-3.42)	.70	0.43 (0.16-1.16)	.10
Base of tongue primary	2.31 (0.72-7.40)	.16	1.06 (0.45-2.5)	.89
Tonsil primary	0.34 (0.09-1.28)	.11	0.59 (0.25-1.40)	.23
T stage ≥3	1.10 (0.16-7.52)	.93	5.52 (2.35-12.97)	<.001
N stage ≥2c	0.70 (0.12-3.98)	.68	1.71 (0.69-4.24)	.25
Human papillomavirus–positive	0.13 (0.02-0.92)	.04	0.16 (0.05-0.49)	.001
Primary tumor resection	0.28 (0.07-1.21)	.09	0.20 (0.06-0.68)	.01
Radiotherapy de-escalated to 30 Gy	0.25 (0.06-1.08)	.06	0.46 (0.16-1.33)	.15
Cisplatin-based chemotherapy	1.51 (0.23-9.81)	.67	0.18 (0.08-0.43)	<.001
Hypoxia-positive pretreatment^c^	NA	NA	1.45 (0.49-4.29)	.50
Hypoxia-positive intratreatment	3.51 (1.05-11.79)	.04	2.66 (1.14-6.19)	.02

Abbreviations: HR, hazard ratio; NA, not applicable.

^a^ Competing risk regression with death as a competing risk.

^b^ Cox regression.

^c^ HR estimation for distant metastasis was not possible due to no events among patients with hypoxia-negative disease pretreatment.

Of 22 deaths, 6 were related to metastatic HNSCC progression. Median (IQR) time to latest clinical follow-up among censored patients was 58 (46-91) months. OS estimates at 5 years were 89.1% (95% CI, 81.8%-97.1%) among patients with persistent intratreatment hypoxia and 95.5% (95% CI, 92.5%-98.7%) among those without (HR, 2.66; 95% CI, 1.14-6.19, *P* = .02) (Figure, C). Advanced age (HR, 1.13; 95% CI, 1.08-1.19; *P* < .001) and T stage 3 or greater (HR, 5.52; 95% CI, 2.35-12.97; *P* < .001) were also associated with worse OS. HPV-positive disease (HR, 0.16; 95% CI, 0.05-0.49; *P* = .001) and cisplatin-based CRT (HR, 0.18; 95% CI, 0.08-0.43; *P* < .001) were associated with improved OS (Table 2).

A subset of patients with HPV-positive oropharyngeal primary tumors who received platinum-based chemotherapy (228 patients) was analyzed separately. The HR for DM risk was 2.79 (95% CI, 0.73-10.58). (eFigure 3 in Supplement 1).

## Discussion

There is ample preclinical evidence demonstrating that hypoxia promotes essentially every step of the metastatic cascade through activation of the hypoxia-inducible transcription factors.^5,6^ In the context of localized disease, a hypoxic tumor microenvironment promotes immune evasion and epithelial-mesenchymal transition, which are precursors for metastasis.

In the clinical management of HNSCC, FMISO PET has been incorporated as a noninvasive method to assess tumor hypoxia and is associated with locoregional recurrence.^14,15,16,17,18^ However, there are limited data supporting its association with DM^19,20^ despite the underlying biological rationale. Because DM is a relatively rare event in HNSCC, a major challenge has been accumulating a sufficient sample size to address this question. Most prospective clinical series exploring the role of FMISO PET in HNSCC have been small, typically ranging from 15 to 53 patients.^14,15,16,17,19,20^

In contrast, in this cohort study, we have compiled a prospective series of 281 patients to evaluate whether tumor hypoxia on FMISO PET is associated with DM risk after CRT for HNSCC. Persistent intratreatment hypoxia was associated with increased DM risk. Conversely, no patients with hypoxia-negative disease before CRT experienced DM. Poorer OS was associated with persistent intratreatment hypoxia but not with pretreatment hypoxia. Compared with prior studies focusing on pretreatment hypoxia,^19,20^ this analysis suggests that intratreatment hypoxia is of major clinical importance.

While there is an established but small-magnitude association of stage with DM risk,^1,2^ hypoxia had a larger magnitude of association with DM than T or N stage in this study. Because most patients had T stage 2 or less and N stage 2b or less, it is unclear if the results can be extrapolated to more advanced disease. In future studies, which would ideally include a greater proportion of locally advanced disease and a greater number of DM events, stage and tumor hypoxia should be evaluated as independent risk factors for DM. Nevertheless, it is notable that hypoxia was associated with increased DM risk even in a predominantly early-stage cohort.

Higher rates of DM among patients with hypoxia-positive disease are expected to translate to worse OS. Given the small number of DM events in a predominantly early-stage cohort, a minority of deaths were related to DM progression. Nevertheless, we observed a signal for worse OS among patients with intratreatment hypoxia.

Prior work has shown that the lack of hypoxia on FMISO PET can be used to select patients for safe, effective de-escalation of CRT. In a series of prospective clinical trials,^16,17,18^ patients with HPV-positive HNSCC who were negative for tumor hypoxia 1 to 2 weeks after initiating CRT were eligible for de-escalated CRT over 3 weeks (30 Gy) compared with standard CRT over 7 weeks (70 Gy). With this selective de-escalation strategy, no differences in progression-free survival or OS were observed.^18^ Conversely, the current study shows that persistent tumor hypoxia on FMISO PET is a biomarker of DM risk and may guide patient selection for escalated therapeutic strategies, including novel systemic therapy regimens. Pembrolizumab has shown efficacy in treating metastatic HNSCC, independent of cytotoxic chemotherapy,^4^ and may reduce DM risk in the upfront setting, for a well-selected high-risk patient population. Ultimately, both the presence and absence of tumor hypoxia may be used as biomarkers to guide individualized therapy.

### Limitations

Our study is not without limitations. Despite the large size of this dataset, there were still few DM events. This limited the ability to assess hypoxia as an independent risk factor in multivariable and subgroup analyses. Additionally, treatment strategies were heterogenous. Patients with and without primary tumor resection were included, as were patients who received standard and de-escalated CRT.

## Conclusions

In this cohort study using pooled analysis of prospective nonrandomized clinical trials, we evaluated tumor hypoxia on FMISO PET as a biomarker of DM risk after CRT for HNSCC. Persistent hypoxia during CRT was associated with increased risk of DM. Conversely, all patients with hypoxia-negative disease before CRT remained free of DM. These findings suggest that tumor hypoxia on FMISO PET may serve as a biomarker of DM risk.

## Notes

Supplement 1. **eFigure 1.** Flow Diagram Illustrating the Number of Patients Included From Each of the 2 Prospective Clinical Trials **eFigure 2.** Examples of FMISO PET in Evaluating Hypoxia of Oropharyngeal Tumors **eTable.** Characteristics of 12 Patients Who Experienced DM **eFigure 3.** Distant Metastasis and Overall Survival After Chemoradiotherapy by Pretreatment and Intratreatment Hypoxia Status for a Subgroup of Patients With HPV-Positive Disease

Supplement 2.
Data Sharing Statement
