# Supplementary material for: Tumor Hypoxia on 18F-fluoromisonidazole Positron Emission Tomography and Distant Metastasis From Head and Neck Squamous Cell Carcinoma
Source: JAMA Netw Open. 2024 Sep 30;7(9):e2436407. doi: 10.1001/jamanetworkopen.2024.36407 (PMC11443350; doi:10.1001/jamanetworkopen.2024.36407)
Supplement: Supplement 1. — eFigure 1. Flow Diagram Illustrating the Number of Patients Included From Each of the 2 Prospective Clinical Trials eFigure 2. Examples of FMISO PET in Evaluating Hypoxia of Oropharyngeal Tumors eTable. Characteristics of 12 Patients Who Experienced DM eFigure 3. Distant Metastasis and Overall Survival After Chemoradiotherapy by Pretreatment and Intratreatment Hypoxia Status for a Subgroup of Patients With HPV-Positive Disease [file jamanetwopen-e2436407-s001.pdf]

Supplemental Online Content

Gui C, Wray R, Schöder H. Tumor hypoxia on FMISO positron emission tomography and distant metastasis from head and neck squamous cell carcinoma. *JAMA Netw. Open*. 2024;7(9):e2436407. doi:10.1001/jamanetworkopen.2024.36407

eFigure 1. Flow Diagram Illustrating the Number of Patients Included From Each of the 2 Prospective Clinical Trials

eFigure 2. Examples of FMISO PET in Evaluating Hypoxia of Oropharyngeal Tumors

eTable. Characteristics of 12 Patients Who Experienced DM

eFigure 3. Distant Metastasis and Overall Survival After Chemoradiotherapy by Pretreatment and Intratreatment Hypoxia Status for a Subgroup of Patients With HPV-Positive Disease

This supplemental material has been provided by the authors to give readers additional information about their work.

© 2024 Gui C et al. *JAMA Netw. Open.*

eFigure 1. Flow Diagram Illustrating the Number of Patients Included from Each of the 2 Prospective Clinical Trials

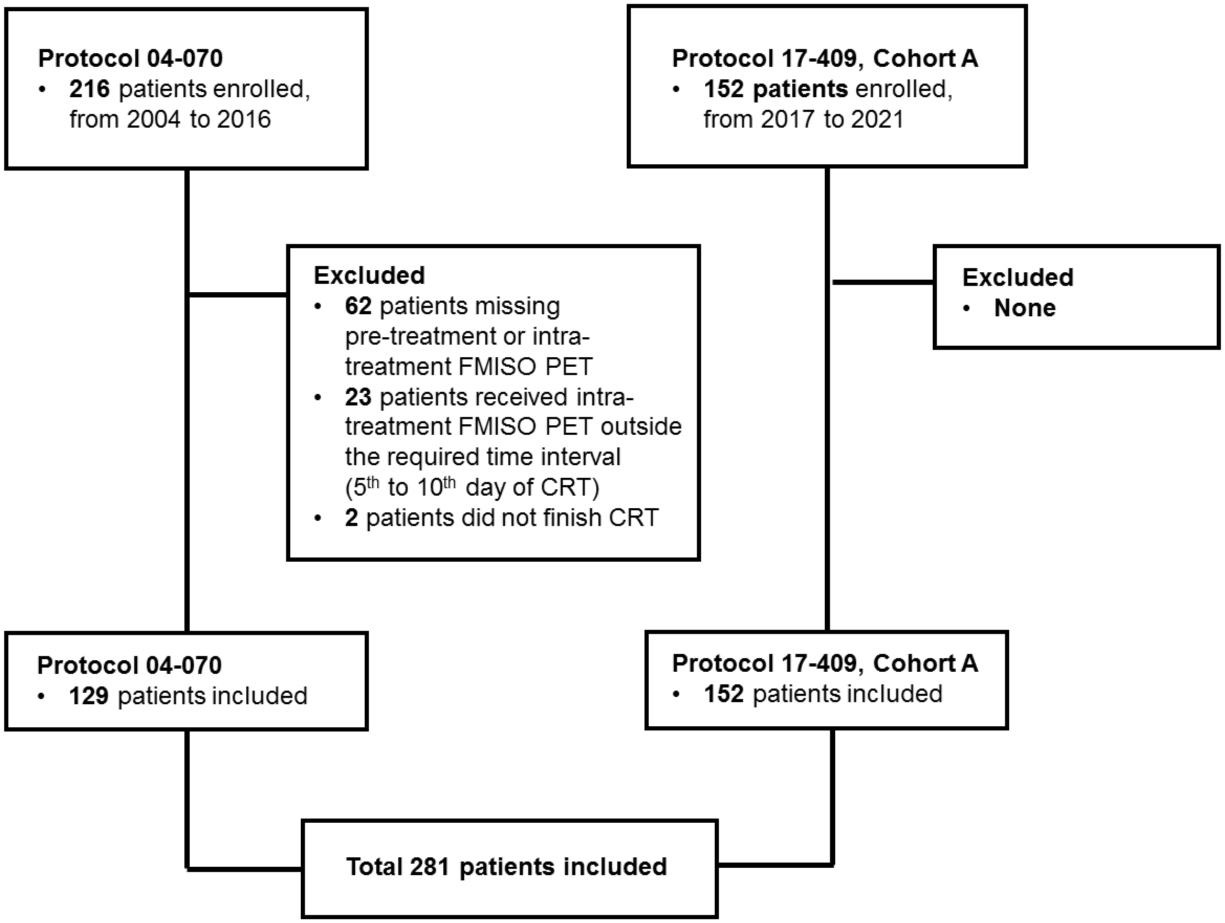

© 2024 Gui C et al. *JAMA Netw. Open.*

eFigure 2. Examples of FMISO PET in Evaluating Hypoxia of Oropharyngeal Tumors

The figure shows examples demonstrating hypoxia negativity before and during treatment (A), hypoxia positivity before treatment that resolved to hypoxia negativity during treatment (B), and persistent hypoxia positivity before and during treatment (C).

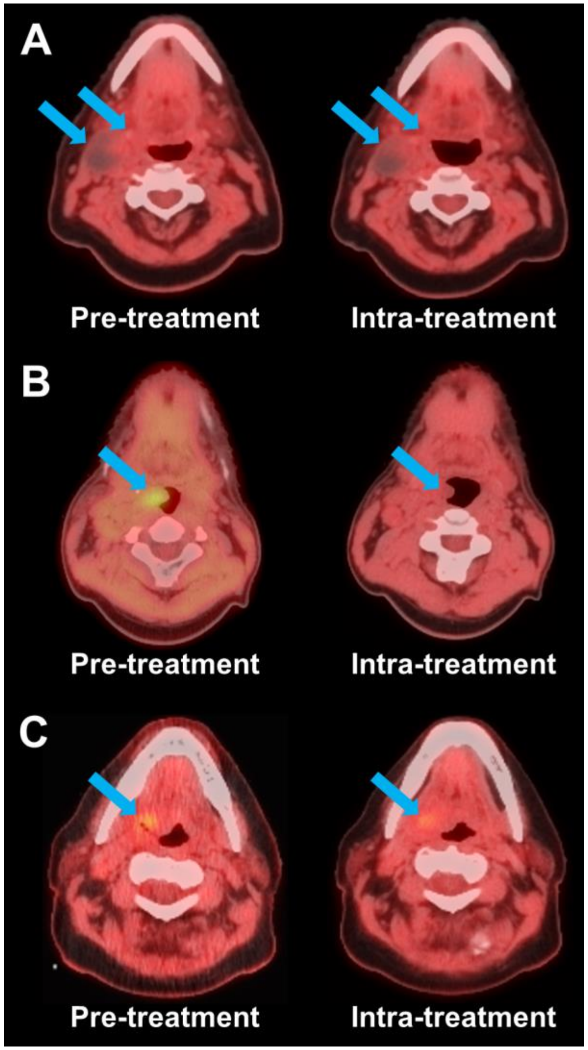

© 2024 Gui C et al. *JAMA Netw. Open.*

eTable. Characteristics of 12 Patients Who Experienced DM

Patient identifier	Pre-treatment hypoxia	Intra-treatment hypoxia	Primary site	HPV-associated	T stage	N stage	Systemic therapy regimen	Time to DM after definitive therapy (months)	Locoregional recurrence	Timing of locoregional recurrence
1	Positive	Positive	BOT	Yes	T3	N2b	Platinum-based	11.6	Yes	Before DM
2	Positive	Positive	Tonsil	Yes	T2	N2b	Platinum-based	9.4	No	NA
3	Positive	Negative	Tonsil	Yes	T1	N2b	Platinum-based	80.1	Yes	Before DM
4	Positive	Negative	BOT	Yes	T2	N2a	Platinum-based	9.9	Yes	Before DM
5	Positive	Positive	BOT	Yes	T1	N2b	Platinum-based	26.7	No	NA
6	Positive	Positive	Larynx	No	T1	N2b	Cetuximab only	2.7	Yes	Concurrent with DM
7	Positive	Negative	BOT	Yes	T2	N2b	Platinum-based	7.7	No	NA
8	Positive	Positive	Larynx	No	T2	N2b	Platinum-based	3.6	No	NA
9	Positive	Negative	BOT	Yes	T2	N2b	Platinum-based	87.4	No	NA
10	Positive	Positive	BOT	Yes	T2	N2b	Platinum-based	13.8	No	NA
11	Positive	Negative	Tonsil	Yes	T3	N2c	Platinum-based	23.3	No	NA
12	Positive	Negative	BOT	Yes	T2	N2c	Platinum-based	19.5	Yes	Before DM

BOT: base of tongue
CRT: chemoradiotherapy
DM: distant metastasis
HPV: human papillomavirus

© 2024 Gui C et al. *JAMA Netw. Open.*

eFigure 3. Distant Metastasis and Overall Survival After Chemoradiotherapy by Pretreatment and Intratreatment Hypoxia Status for a Subgroup of Patients With HPV-Positive Disease

This supplemental figure describes a subset of the overall study cohort. Patients with HPV-positive oropharyngeal primary tumors treated with platinum-based chemoradiation (228 patients) are included.

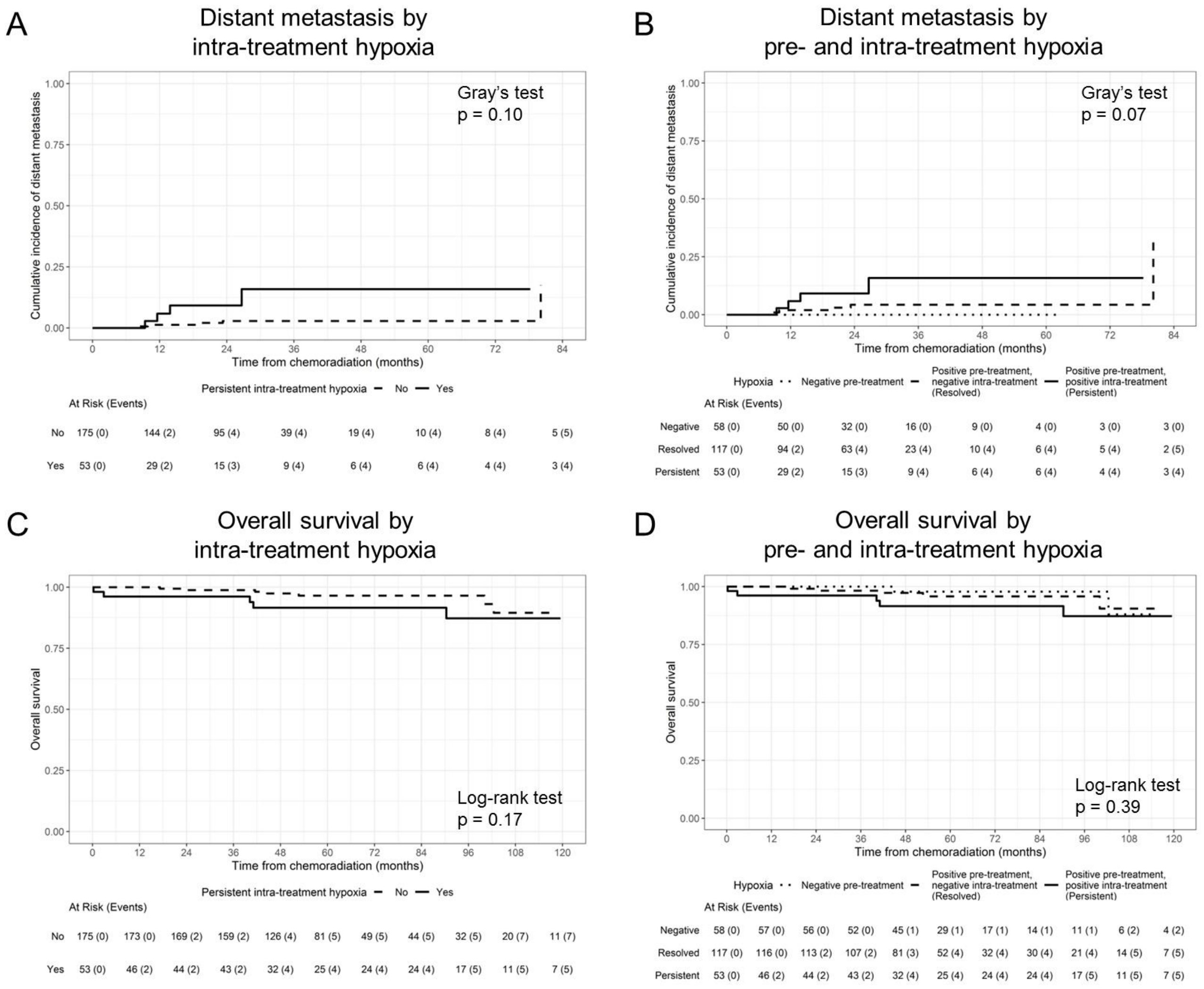

© 2024 Gui C et al. *JAMA Netw. Open.*

© 2024 Gui C et al. *JAMA Netw. Open.*
