# Supplementary material for: Tumor Hypoxia on 18F-fluoromisonidazole Positron Emission Tomography and Distant Metastasis From Head and Neck Squamous Cell Carcinoma
Source: JAMA Netw Open. 2024 Sep 30;7(9):e2436407. doi: 10.1001/jamanetworkopen.2024.36407 (PMC11443350; doi:10.1001/jamanetworkopen.2024.36407)
Supplement: Supplement 2. — Data Sharing Statement [file jamanetwopen-e2436407-s002.pdf]

Data Sharing Statement

Gui. Tumor Hypoxia on FMISO Positron Emission Tomography and Distant Metastasis from Head and Neck Squamous Cell Carcinoma. *JAMA Netw Open*. Published September 30, 2024. doi:10.1001/jamanetworkopen.2024.36407

Data
Data available: No
